# State of the Art of Genomic Technology in Toxicology: A Review

**DOI:** 10.3390/ijms24119618

**Published:** 2023-06-01

**Authors:** Rogelio Recio-Vega, Rolando Adair Facio-Campos, Sandra Isabel Hernández-González, Edgar Olivas-Calderón

**Affiliations:** 1 Environmental Health Unit-PEÑOLES, Torreòn 27220, Mexico; rrecio@yahoo.com; 2 Laboratory of Environmental Health, School of Chemical Sciences, Juarez University of Durango State, Gomez Palacio 35010, Mexico; rfaciocampos@gmail.com (R.A.F.-C.); sandraisabel_70@hotmail.com (S.I.H.-G.)

**Keywords:** genomic technology, omics, toxicology

## Abstract

The rapid growth of genomics techniques has revolutionized and impacted, greatly and positively, the knowledge of toxicology, ushering it into a “new era”: the era of genomic technology (GT). This great advance permits us to analyze the whole genome, to know the gene response to toxicants and environmental stressors, and to determine the specific profiles of gene expression, among many other approaches. The aim of this work was to compile and narrate the recent research on GT during the last 2 years (2020–2022). A literature search was managed using the PubMed and Medscape interfaces on the Medline database. Relevant articles published in peer-reviewed journals were retrieved and their main results and conclusions are mentioned briefly. It is quite important to form a multidisciplinary taskforce on GT with the aim of designing and implementing a comprehensive, collaborative, and a strategic work plan, prioritizing and assessing the most relevant diseases, so as to decrease human morbimortality due to exposure to environmental chemicals and stressors.

## 1. Introduction

**Figure 1:**
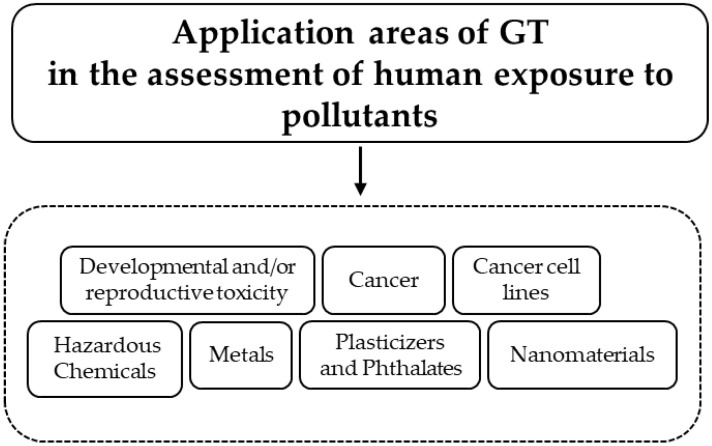
Appropriate application for GT.

The rapid growth of genomic techniques has revolutionized and impacted the knowledge in medical science considerably and positively, and toxicology has ushered it into a “new era”: the era of GT (Figure 1).

**Figure 2:**
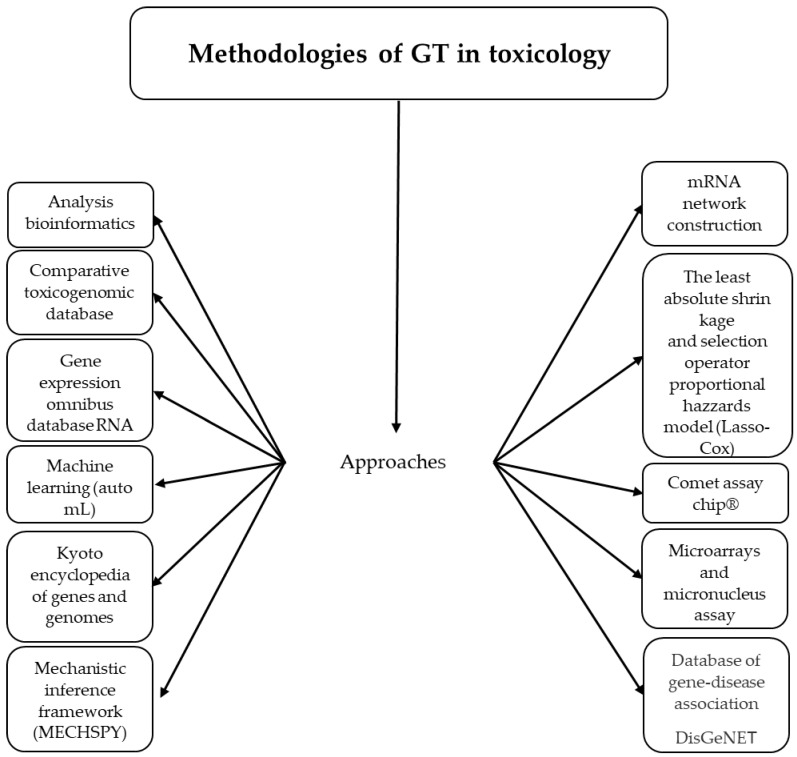
Tools of GT used in medical sciences and toxicology.

Indeed, with the use of the omics technologies, toxicology has evolved impressively, and it is now possible to analyze the whole genome, elucidate the toxicant pathways, know the modes of action of toxicants, recognize the association between toxic stressors and disease susceptibility, identify early biomarkers of disease, exposure, and risk, determine drug targets, evaluate exposure assessment, hazard screening, cross-species extrapolation, the dose–response relationship, developmental exposure, and the gene response to toxicants and environmental stressors, and to determine specific profiles of gene expression through various tools [1] (Figure 2).

The incredible amounts of information generated using these omics approaches alone or conjointly (Table 1) can be employed to design integral health programs with the aim of decreasing human morbimortality due to the side effects of exposure to environmental chemicals and stressors.

In GT, the most common omics and frequently used are transcriptomics, proteomics, and metabonomics or metabolomics. Transcriptomics is utilized to elucidate the mechanism of action. Proteomics measures proteins expressed in the presence or absence of toxicity to show the affected signaling pathways in order to determine possible biomarkers and to evaluate toxicity after exposure; it is also used for the monitoring of disease development, early diagnosis, and prognosis [2]. Metabolomics is related to the study of the metabolites present in cells, tissues, organisms, and fluids that may establish variations in the levels of small endogenous molecules as changes in a sequence of key metabolic events, such as “metabolite fingerprints”, which will help to diagnose and define the ways in which specific chemicals, environmental exposures, or stressors cause diseases [17]. Another interesting omics approach is epigenetics, which plays a key role in individual development and in the appearance of diseases through the regulation of gene expression, including the inhibition of transposon, affecting promoters, and regulating chromatin states. The majority of studies on epigenetic modification includes DNA methylation, histone modification, and noncoding RNA [18]. However, the application of metabolomic technologies is somewhat restricted worldwide, due to speed, cost, and data quality [19]. In addition to omics technologies, machine learning (ML) and artificial intelligence (AI) approaches are increasingly applied in different subject areas of toxicology, and their applications are employed mainly for the prediction and evaluation of the chemical, toxicokinetic, and toxicity properties of xenobiotics.

The aim of this work was to compile and narrate the recent research on GT during the last 2 years. 

## 2. Methods

According to the Preferred Reporting Items for Systematic Reviews and Meta-Analyses (PRISMA) guidelines [20], a review of the published literature were performed. Reviewed papers were grouped according to their main research area and results are mentioned concisely and briefly. A search of the literature was conducted using PubMed (https://pubmed.ncbi.nlm.nih.gov) and Medscape (https://www.medscape.com) databases filtered by publication date from 15 January 2020 to 5 September 2022. Keywords for searching were, omics, GT, and toxicology. Relevant articles were published in peer-reviewed journals. Selected articles were recent, and original and updated systematic reviews only if described topics related to molecular technology applied in toxicology. Selected articles were reviewed for all authors, including toxicants as metals, environmental pollutants, and hazardous chemicals where gene techniques were used. Two hundred and ten articles were found and only eighty-nine were included in this work.

## 3. Developmental and/or Reproductive Toxicity

Exposure to xenobiotics during pregnancy may cause developmental and/or reproductive toxicity; therefore, it is most important to identify those chemicals of high toxicity. Tung et al. [21] proposed a genetic-algorithm-based method to develop a weight-of-evidence (WoE) model and the authors identified 26 chemicals of concern with high toxicity, among which 13 chemicals have been reported to be developmentally or reproductively toxic.

Spontaneous abortion or miscarriage is defined as the loss of a pregnancy of a gestation of less than 20 weeks. The American College of Obstetricians and Gynecologists estimates that this is the most common form of loss of pregnancy. It is estimated that as many as 26% of all pregnancies and up to 10% of clinically recognized pregnancies end in miscarriage [22]. Risk factors for miscarriage are poorly studied. Harris et al. [23] utilized miscarriage (MeSH: D000022) and chemical gene lists from the CTD in human, mouse, and rat. The authors observed that several chemical gene sets (parathion, cadmium, naphthalene, carbon tetrachloride, arsenic, lead, dieldrin, and atrazine) were highly enriched for miscarriage genes.

Testes are sensitive to tissue disruption, as they contain multiple cell types under constant division and/or maturation, and it has been reported that 2,3,7,8-tetrachlorodibenzo-p-dioxin (TCDD) exposure may increase the susceptibility. Haimbaugh et al. [24] compared the results and conditions of two methods of transcriptomic examination on adult zebrafish testes exposed to TCDD during sexual differentiation. The results of these authors revealed that TCDD-related genes may be overlooked by scRNA-Seq; however, this underdetection effect is not mediated by exposure to this compound. 

Phthalates or phthalate esters are esters of phthalic acid. They are mainly used as plasticizers, to make plastics more durable. Chronic exposure to phthalates will adversely influence the endocrine system and the functioning of multiple organs, involving negative long-term impacts on the success of pregnancy, child growth and development, and reproductive systems in the early stages of human life in men [25]. Baralić et al. [26] determined and compared the capacity of bis (2-ethylhexyl) phthalate (DEHP), dibutyl phthalate (DBP), bisphenol A (BPA), and their mixture to produce testicular toxicity. The authors found effects on metabolism, the AhR pathway, apoptosis, and oxidative stress (OS) being singled out as the most probable mechanisms involved in the subacute DEHP–DBP–BPA mixture of testicular harm, while the effect on OS parameters was confirmed by an in vivo experiment.

Arsenic (As) poisoning and its possible reproductive functional damage comprise a global environmental interest. Recent studies showed that spermiogenesis tends to be a major target process in arsenic-induced male infertility; however, the underlying mechanisms of this are not fully understood [27]. Some environmental pollutants give rise to OS and DNA damage by inducing epigenetic alterations [28]. Lu et al. [29] studied the impact of paternal exposure to arsenic on the human sperm DNA methylation status of imprinting genes. The authors found that paternal nonoccupational exposure to arsenic induces the altered DNA methylation status of *Meg3* in human sperm DNA. These findings would implicate the sensitivity of the sperm epigenome in terms of environmental pollution.

Polycystic ovary syndrome (PCOS) is the major endocrinopathy among reproductive-aged women. It affects 4–20% of women of reproductive age worldwide, and it has been associated with exposure to different contaminants [30,31]. Zeng et al. [32] downloaded, from the Gene Expression Omnibus database, the RNA or miRNA expression-profile datasets of patients with PCOS. These authors demonstrated that the protein–protein interaction network revealed that MAP3K14 and TXNIP could keep interacting with both hub genes *PLK1* (degree = 21) and *TLR1* (degree = 18), respectively. 

Endocrine-disrupting compounds (EDC), metals, and metalloids are a persistent threat to humans and wildlife due to their ability to interfere with endocrine signaling pathways. To improve the identification of EDC hazards employing GT data, Sakhteman et al. [33] developed a genomic-oriented data space for profiling the molecular activity of EDC in silico and for creating predictive models that recognize and prioritize EDC.

## 4. Cancer

Cancer is a complicated disease in which cancer cells express epigenetic and transcriptomic mechanisms to commence tumor initiation, progression, and survival. Characterizing the interaction of the epigenome and the transcriptome is essential for understanding cancer cell line behavior, but also for future disease treatment and drug development [34]. Breast, cervical, and ovarian cancers are the three most common malignancies in women. Gong et al. [35] identified the associations between chemical exposure and the aberrant expression, repression, or mutation of genes related with the more frequent cancers in women. The authors identified five chemicals (NSC668394, glafenine, methylnitronitrosoguanidine, fenofibrate, and methylparaben) that were associated with the incidence of breast cancer and cervical cancer. Liu et al. [36] studied the differently expressed RNA binding proteins between four subtypes of breast cancer and normal tissues. These authors concluded that *MRPL12*, *MRPL13*, and *POP1* might act as oncogenes in maintaining cellular viability and stimulating the metastasis of breast cancer cells, involving the possibility of their being designed as biomarkers and/or therapeutic targets for breast cancer.

Renal cell carcinoma (RCC) accounts for more than 400,000 new cases diagnosed and about 180,000 deaths worldwide in 2020 [37]. High blood levels of heavy metals have been associated with a variety of diseases, including cancer. Panaiyadiyan et al. [38] discovered a notably higher concentration of As, Cu, Mn, Cd, Pb, and Hg in the blood of patients with RCC. Meng et al. [39] investigated the role of chromatin accessibility in the development and progression of clear cell renal cell carcinoma (ccRCC). The authors discovered five predictors (*FSCN1*, *SLC17A9*, *ANKRD13B*, *ADCY2*, and *MAPT*), and a prognostic model based on these genes through the least-absolute-shrinkage and selection operator–proportional hazards model (LASSO–Cox) analysis. These authors highlight the important role of chromatin accessibility in ccRCC.

## 5. Cancer Cell Lines in GT

Cancer cell lines are the most used models for studying cancer biology, for validating cancer targets, and for defining drug efficacy. Liu et al. [40] assessed whether transcriptomic data can be utilized to support the assessment of toxicity by means of the comparison of transcriptomic profiles from three cancer lines (HL60, MCF7, and PC3). The authors concluded that the repurposing of existing cancer-related transcript-profiling data entertains great potential for toxicity assessment, particularly in predicting limited-to-drug-induced liver injury.

Schyman et al. [41] noted the expression of several common genes, including *SPP1*, *TNSF18*, *SERPINE1*, *CLDN4*, *TIMP1*, *CD44*, and *LGALS3*, the activation of injury-specific KEGG pathways, and the alteration of plasma metabolites involved in amino acid and bile acid metabolism. The latter are some of the important molecular processes that changed early after thioacetamide exposure and may have a significant impact on the onset of acute liver injury.

HepaRG™ cells are mature hepatocyte-like cells that are metabolically competent cells that express phase I and phase II metabolic enzymes, making them perfect for toxicity testing. In order to identify DNA-damage-inducing (DDI) substances in human HepaRG™ cells, Buick et al. [42] evaluated the efficacy of the flow cytometry in vitro micronucleus (MN) test and the GT-DNA-damage-inducing transcriptomic biomarker. According to these authors, pairing the test techniques allowed for the accurate classification of all five DDI and five non-DDI agents as genotoxic/nongenotoxic and DDI/non-DDI, respectively.

The majority of ToxCast in vitro screening lacks integrated physiological functionality (such as receptor signaling, metabolism). Franzosa et al. [43], in an attempt to integrate this, evaluated, in differentiated HepaRGTM cells, the expression of 93 gene transcripts by qPCR arrays in response to 1060 chemicals. These authors used six transcription factors, including the aryl hydrocarbon receptor, constitutive androstane receptor, pregnane X receptor, farnesoid X receptor, and androgen receptor, as well as peroxisome-proliferator-activated receptor alpha, to quantitatively model chemically induced changes in gene expression.

Different in vitro cell models have been utilized for the study of xenobiotic metabolism; however, human in vitro liver-cell models may yield different results when employed in human liver tissue. Similarly, but now in rat liver tissues, Luijten et al. [44] showed that a relatively small number of matches observed in vitro were also observed in vivo. Human HepaRG hepatocarcinoma cells that had been exposed to several genotoxins were used by Kreuzer et al. [45] to compare transcriptome data, finding that a significant variance was seen in the quantities and identities of differentially expressed genes, even when similar-acting drugs were used in the same cell line. In addition to the previous data, Gupta et al. [46] used RNA-Seq to analyze in vitro liver cell models in comparison with human liver tissue (HepG2, HepaRG 3D iPSC-HLC, PHH, and 3D liver microtissues). These authors found that 3D liver microtissues exhibited a high similarity with in vivo liver, that HepG2 cells had the lowest similarity with human liver tissue, and that HepaRG models, that is, iPSC-HLC and hPCLiS, had a range clearly behind that of the microtissues and PHH, but that revealed a higher similarity to human liver tissue than HepG2 cells. 

Tris (2-ethylhexyl) phosphate (TEHP) is a suspected hepatocarcinogen and an organophosphate flame retardant. Saquib et al. [47] used HepG2 cells treated with TEHP to evaluate 84 genes by qPCR array. The authors found 10 upregulated genes and four downregulated genes belonging to a human cancer pathway. Perkins et al. [48] developed a causal subnetwork of 28 nodes that represents the key event of regenerative proliferation. These authors found that Cyclin D1 was overexpressed after exposures to carbon tetrachloride, Aflatoxin B1, and Thioacetamide, but not in exposures to Diazepam and Simvastatin.

## 6. Hazardous Chemicals

The IARC has categorized formaldehyde (FA) as a Group-I human carcinogen. It is unknown how many individual genes, metabolic pathways, and FA-activating processes work in human cells. Mohanty et al. [49] investigated the toxicogenomic and proteomic alterations in liver of rats fed with FA. Gene Ontology analysis showed that binding, catalysis, and signal transduction were affected in FA-fed rats. Pathway analysis revealed that the formaldehyde exposure activated the PI3K-AKT pathway, leading to the inhibition of caspase activity, and that FA could be less toxic and nontumorigenic at low concentrations. Furthermore, Zhao et al. [50] used genome-wide CRISPR screening for loss-of-function to find modulators of FA toxicity in the human hematopoietic K562 cell line. The authors identified several potential genes that, when disrupted, enhanced sensitivity to FA (such as *ADH5, ESD,* and the *FANC* family) or resistance (such as *FASN* and *KDM6A*). Kang et al. [51] studied the toxic effects of inhaled formaldehyde and identified six upregulated hub genes (*AREG*, *CXCL2*, *HMOX1*, *PLAUR*, *PTGS2*, and *TIMP1*).

U.S. law limits the use of active ingredients in pesticides or biocides for certain major modes of action (MoA) in environmental nontarget organisms. Utilizing the zebrafish embryo model, six nerve- and muscle-targeting insecticides with various MoAs were exposed to a variety of sublethal doses, which Reinwald et al. [52] described as transcriptome responses. There were 222 early responsive genes found, many of which were associated with the following three main processes: (1) the formation and operation of cardiac muscle cells; (2) oxygen transport and hypoxic stress; and (3) the growth and plasticity of neurons. Chlorpyrifos and greater levels of abamectin both elevated the thyroid-related gene *dio3b* and downregulated it. The most commonly differentially expressed genes were those involved in the regulation of heart muscle (*tcap*) and forebrain development (*npas4a*) across all insecticidal treatments. Alugubelly et al. [4] evaluated chlorpyrifos-methyl, which is an organophosphate pesticide used to control insects on fruits, vegetables, and cereal plants, and observed changes in protein expression and in the associated neurotransmitter systems in the adolescent brain that are altered by early developmental exposure to chlorpyrifos; in addition, even at low levels of chlorpyrifos, it inhibits fatty acid amide hydrolase, but not acetylcholinesterase.

Another dangerous substance is nitrotoluene, which is widely used in photography production and in colorant chemicals; additionally, it is explosive. Gust et al. [53] channeled the adverse outcome pathways (AOP) concept to test the hypothesis that the inhibition of PPARα signaling in nitrotoluene exposures impacted lipid metabolic processes. These authors found the inhibition of nuclear transactivation for genes controlling lipid metabolism and ketogenesis, the inhibition of fatty acid beta-oxidation and ketogenesis dynamics, and a negative energy budget.

Exposure to bisphenol A (BPA) has been related to adverse effects on the reproductive system and during development. Li et al. [54] evaluated BPA’s possible mode of action (MOA) in terms of reproductive/developmental toxicity, neurological toxicity, and proliferative effects on the mammary gland and the prostate that may be associated with carcinogenesis. Based on their findings, it is possible that target genes for estrogen receptor 1, estrogen receptor 2, mitogen-activated protein kinase 1 (MAPK1), MAPK3, BCL2 (an apoptosis regulator), caspase 3, BAX, and androgen receptor, as well as AKT serine/threonine kinase 1, are all related. The common phenotypes with various target organs may include apoptosis, cell proliferation, the biosynthesis of testosterone, and the biosynthesis of estrogen. The estrogen signaling pathway and cancer-related pathways may also be involved in the Kyoto Encyclopedia of Genes and Genomes (KEGG) pathways of the BPA-induced activity. 

Among the main pollutants found in the air are the organic pollutants denominated as polycyclic aromatic compounds (PACs), which are associated with cardiovascular-, immune-, and reproductive-system toxicity. Halappanavar et al. [55] revealed that nitrated and oxygenated polycyclic aromatic hydrocarbons (PAHs) drive the response at lower concentrations, while specific PAHs drive the response at the highest concentration tested. On the other hand, Kim et al. [56] employed primary human nasal epithelial cells (PHNEC) to identify signaling changes brought on by exposure to diesel exhaust particles (DEP). Major signaling changes in PHNEC brought on by DEP exposure were predicted to stimulate pathways associated to inflammation and immune response that are mediated by TNF. Key hub genes in the anticipated pathway, including *CSF3*, *CXCL8*, *MMP1*, and *VEGFA*, have been identified.

In addition, Đukić-Ćosić et al. [57] investigated the connection between the main air pollutants (sulfur dioxide (SO), carbon monoxide (CO), particulate matter (PMx), nitrogen dioxide (NO_2_), and ozone (O_3_)) and COVID-19 and discovered that SO_2_, CO, PMx, NO_2_, and O_3_ interacted with 6, 6, 18, 9, and 12 COVID-19-related genes, respectively. This is because it has been suggested that air pollution may have an impact on COVID-19 transmission, severity, and death rate. IL-10, IL-6, IL-1B, and TNF are present in all pollutants involved in the majority (77.64%) of physical interactions. 

The risk assessment of mixtures requires the efficient integration of in vivo, in vitro, and in silico data with the data of epidemiology and human studies. Ruiz et al. [58] studied toluene, ethylbenzene, and xylene (*TEX*) gene–disease associations utilizing CTD pathway analysis, and the authors published microarray data from human gene-expression changes. The results of these authors reveal that 236 of the genes expressed were common between short- and long-term exposures. These genes could be central for the interconnecting biological pathways potentially stimulated by *TEX* exposure, likely related to respiratory and neurological diseases. 

Graphene comprises a monolayer of carbon atoms tightly bound in a hexagonal lattice, and it commands a wide use in industry. Poulsen et al. [59] explored the transcriptomic differences in female C57BL/6 mouse lung and liver after pulmonary exposure to two graphene-based materials (GBM). The most significant alterations were seen in the acute-phase response and hepatic lipid homeostasis, both of which were highly stimulated by graphene oxide exposure. In contrast to both GBM, exposure to graphene-oxide also specifically caused changes in the transcriptome linked to fibrosis. These changes were attributed to the generation of reactive oxygen species (ROS) and genotoxicity.

Dihydroxyacetone (DHA) is a skin-coloring agent, and it is used as a treatment for patients with vitiligo. After its contact with the epidermis, it penetrates beyond the stratum corneum to the keratinocytes; and its toxic effects have not yet been elucidated. Striz et al. [60] exposed Normal Human Epidermal Keratinocyte (NHEK) cells to DHA, discovering that primary keratinocytes are cytotoxic to DHA above 25 mM. Genotoxicity was only found at cytotoxic concentrations, probably due to DNA damage that is not biologically relevant, whereas subtoxic dosages caused alterations in gene expression and glycation.

Carboxylic acids are found widespread in nature, often combined with other functional groups. Some carboxylic acids, such as valproic acid (VPA), which is used to treat some forms of seizures in rats, are known to produce hepatic steatosis and significant birth abnormalities, particularly damaging the brain and spinal cord. Vrijenhoek et al. [61] examined the transcriptome responses of primary human hepatocytes (PHH) to 18 structurally different VPA analogs. According to the authors, active carboxylic acids influence the regulation of free fatty acid production and stress pathway responses.

## 7. Metals

Cadmium (Cd) is a toxic metal that induces the dysregulation of divalent ion homeostasis, and the mechanisms of its recognized human carcinogenicity remain under investigation. Oldani et al. [62] isolated and characterized Cd-transformed differentially expressed genes (DEGs) by whole genome microarray and bioinformatics analysis. These authors reported that only 34 genes in common DEGs are found in cells from all foci, and among these, only four genes are jointly upregulated (*Ccl2*, *Ccl5*, *IL6*, and *Spp1*), all of these responsible for cytokine/chemokine coding. On the other hand, Forcella et al. [63] searched for any nonspecific molecular fingerprints using transcriptome data from cell models that represented the three main Cd targets: lung (*A549*), hepatic (*HepG2*), and neuronal (*SH-SY-5Y*) cells. The authors discovered dysregulated genes associated with metabolic and detoxifying processes (as determined by gene ontology and KEGG). 

To discriminate between metal and nonmetal toxicants, we can create prediction models by identifying and using sensitive and specific gene markers. Yu et al. [64] proposed that employing a microarray classifier analysis can not only uncover significant biomarkers to understand the common and underlying harmful processes generated by metals, but also construct diagnostic classifiers for identifying an exact metal contaminant.

Rehman et al. [65] developed a comprehensive genome-wide investigation to examine the effects of many significant single nucleotide polymorphisms related to As exposure on the methylome in order to comprehend how exposure to As modifies gene expression through epigenetic alterations. The hypermethylation of *CSE1L* and *TRRAP* revealed a distinct route (direct P53 effector) linked to the individual DNMT1a polymorphism and provided the first proof of As-associated DNA methylation in connection to the expression of the *ATR*, *ATF7IP*, *TPM3*, and *UBE2J2* genes. Fu et al. [16] discovered that cells treated with As^3^ had a significant suppression of all metabolites in the mitochondrial tricarboxylic acid cycle. These findings could lead to more accurate descriptions of the role of As in molecular carcinogenesis. With respect to malignant cell transformation by As, Shukla et al. [13] reported that stem cell activators may be crucial in enabling As-exposed cells to obtain a survival edge and reprogramming healthy epithelial cells into a cancer stem cell phenotype. Rehman et al. [66] performed whole-genome gene-expression analysis in the blood of subjects exposed to arsenic (As) using microarrays. Their findings validated crucial signaling, growth factor, cancer, and other disease-related pathways that have been linked to elevated levels of As exposure. The genes *NDUFV3*, *IKBKB*, *IL6R*, *ADIPOR1*, *PPARA*, *OGT*, and *FOXO1* looked to be highly downregulated, which further appeared to dramatically increase the risk of NAFLD and diabetes. The combined toxicity of hexaconazole and As was assessed by Sun et al. [7], and these authors concluded that As mainly altered amino-acid-related metabolites and pathways related to nerve disease, energy deficiency, and liver functional disorder.

Furthermore, As toxicity may result from epigenetic dysregulation, which includes alterations in DNA methylation (DNAm). Combining the results from four EWAS using a harmonized data processing and analysis pipeline and performing a meta-analysis, Bozack et al. [9] found that lysosome, autophagy, and mTOR signaling, AMPK signaling, and one carbon pool by folate were among the KEGG pathways associated with As, concluding that standardizing analytical pipelines can help in identifying biologically meaningful signals. Cognitive decline may result from As epigenomic changes in pathophysiological neural changes, including histone–methylation profile.

Age-related impairment of activities of daily living (ADL) and disability have been linked to higher exposures to manganese, copper, arsenic, and cadmium. Potential mechanisms of DNA methylation for various heavy metals and potential biomarkers of ADL were highlighted by the epigenome-wide association of DNA methylation [11]. 

Parkinson disease (PD) is a movement disorder defined by the gradual degeneration of dopaminergic neurons in the substantia nigra pars compacta, leading to dopamine insufficiency in the striatum. A number of genetic changes have also been connected to heavy metal exposure, even though the majority of PD cases are sporadic. Wang et al. [8] found a significant reduction in docosahexaenoic acid, glycoursodeoxycholic acid, and arachidonic acid, and a significant induction of p-cresol sulfate and phenylacetyl-l-glutamine. In addition, through Gene Ontology (GO) analysis, these authors highlighted the role in brain functions and neurodegenerative diseases such as Parkinson disease and low cognition in subjects with high plasma Pb levels. In order to measure metal-induced cognitive impairment, the authors of this paper offer unique insights into the utilization of plasma metabolites. Cunha Nascimento et al. [14] assessed the effects of maternal MeHg exposure on the modulation of protein biomarkers in the parotid (PA), submandibular (SM), and sublingual (SL) glands of offspring rats. The results showed significant changes in the proteomic profiles of the PA, SM, and SL glands, the latter of which are linked to cytoskeletal components, tissue morphogenesis, and response to stimulus and stress.

The toxicological profile of lanthanides, a metal widely used in multiple industries such as optoelectronics and healthcare, has been incompletely characterized. Pallares et al. [67] assessed the potential toxicity mechanisms in the lanthanide series utilizing a functional GT approach in baker’s yeast. The protein–protein network analysis indicated that a yeast response to lanthanides that relied on proteins that participate in regulatory paths used for calcium and lanthanide toxicity included the disruption of biosynthetic pathways by enzyme inhibition.

## 8. Plasticizers and Phthalates 

Plastics are composed of many additives, such as plasticizers, stabilizers, antioxidants, flame retardants, and others, which can affect the human health. Various genes are affected by plastic additives and are related to apoptosis, cell death, proliferation, differentiation, immunity, and insulin-related processes, and are mainly associated with cancer, mental disorders, diabetes mellitus type II, and obesity. Sendra et al. [68] employed an integrative approach in an attempt to identify the genes, biological processes, molecular functions, and diseases linked with exposure to these compounds and found a strong interconnection among the top 50 genes modulated by plastic additives. 

Acetamide is the simplest amide derived from acetic acid and it is utilized as a plasticizer, as an industrial solvent, and it has been detected in common foods. It is classified as a group-2B (possible human carcinogen). Nault et al. [69] identified 1110 and 1814 differentially expressed genes in male and female rats, respectively, concluding that acetamide is most likely acting through a mitogenic MoA.

Exposure to phthalate and bisphenol A have been associated with asthma and other diseases. Baralić et al. [26] explored the mechanisms of bis (2-ethylhexyl) phthalate (DEHP), dibutyl phthalate (DBP), and bisphenol A (BPA) mixture-induced asthma development. A total of 24 DEHP, DBP, and BPA asthma-related genes were discovered by these researchers, showing the three most likely mechanisms: apoptosis, inflammation, and oxidative stress, demonstrating significant redox status alterations in their in vivo experiment. Bisphenol S (BPS) has been introduced into the industry as a safer alternative to bisphenol A. The mechanisms underlying BPS toxicity may be related to the chemical properties of BPS in the human body, including interactions with estrogen receptors and binding to DNA and some proteins, subsequently including the exertion of OS [70]. Ao et al. [12] investigated the potential consequences and underlying mechanisms of BPS exposure on human colon mucosal epithelial cells (NCM460) and they came to the conclusion that BPS may upset the balance of the gut–brain axis, resulting in the production of inflammatory cytokines and the destruction of tight junctions in NCM460 cells.

Another hazardous chemical is dibutyl phthalate (DBP), a chemical that is continuously present in many consumer items and is thus present in the general population. Although DBP predominantly affects the endocrine and reproductive systems, it can also have an impact on the vasculature system’s functionality. Stanic et al. [71] integrated the toxicogenomic data of human vascular endothelial cells (ECs) in order to infer pathways, molecular activities, biological processes, and human diseases related to DBP exposure. Nine genes, including six members of the integrin family, *VCAM1*, *ICAM1*, and *MMP2*, were found to be affected by DBP exposure, according to these authors, who concluded that changes in the DBP-affected genes could affect the extracellular matrix and the binding of molecules and cells to ECs, changing cell adhesion and migration.

## 9. Nanomaterials

Omics methods have been used to investigate the toxicity of nanomaterials (NMs) in order to learn more about their biological impacts. Epigenetic modifications have been linked to a number of NMs. The novel omics techniques might help exclude or reduce the handling of hazardous NMs in the workplace and enable the adoption of regulations to safeguard human health as researchers seek to understand the molecular alterations involved in NM toxicity [72]. A sequential lineal adverse outcome pathway (AOP) landscape is defined by the integrative investigation of the cellular and molecular processes of nanotoxicity toward the discovery of related adverse consequences. NMs have distinctive characteristics and particular toxicological problems that may constitute uncharted AOP environments [73]. Saarimäki et al. [74] curated and reported a collection of homogenized transcriptomics data from human, mouse, and rat ENM exposures in vitro and in vivo in an effort to improve the fairness of already existing transcriptomic data for engineered nanomaterials (ENM) due to the lack of an easily accessible and reusable transcriptomics data collection for the potential toxicity mechanisms of ENM.

Nickel oxide nanoparticles (NiO-NPs) cause liver damage in both in vitro and in vivo test models, and they have been employed in a number of consumer items. Using high-throughput RNA sequencing (RNA-Seq) and microscopic techniques, Saquib et al.’s [75] toxicogenomic method revealed hepatotoxicity in human hepatocellular carcinoma cells (HepG2). The authors discovered that a treatment with NiO-NPs at a nontoxic dose resulted in a considerable shift in the transcriptome at the mRNA and pathway level, as well as upregulation of hypoxia-inducible transcription factor-1alpha (HIF-1α) and miR-210 microRNA.

Iron oxide nanoparticles (IONPs) could be used in the near future as an alternative in nanomedicine due to their potential applications in tumor therapy, drug delivery, or bioimaging; however, many xenobiotics may be recognized by the immune system and may induce inflammation, hypersensitivity, or anaphylactic shock [76]. In the AKT/mTOR/TFEB signaling pathway, Han et al. [2] evaluated the mechanisms of the toxicity of iron oxide nanoparticles and found 197 upregulated and 75 downregulated proteins that may promote autophagy and lysosomal activation in splenic macrophages.

## 10. Computational Biology/Toxicology

The evaluation of drug toxicity is a very important step in the development of novel drugs. The evaluation of medication safety may be improved in many ways with the application of current developments in machine learning techniques and the quick increase in Big Toxicity Data, including molecular descriptors, toxicogenomics, and high-throughput bioactivity data. Vo et al. [77] and, more recently, Lin and Chou [78] summarized the most notable recent applications of machine learning (ML) and artificial intelligence (AI) techniques in various areas of toxicology, including physiologically based pharmacokinetic (PBPK) modeling, quantitative structure–activity relationship modeling for toxicity prediction, adverse outcome pathway analysis, high-throughput screening, GT, Big Data, and toxicological databases.

After considering different ML algorithms applied in two different splitting strategies, Ochoteco et al. [79] reported that the random forest algorithm can predict proteins in new samples of transcriptomic data with good accuracy. The authors’ proposed preprocessing and model-building scripts can be accessed on GitHub.

One of the most important endpoints for the risk evaluation of food contact chemicals (FCC) is carcinogenicity. Wang et al. [80] suggested a brand-new ML-based weight-of-evidence (WoE) model for placing chemical carcinogenesis at the top of the list. A total of 44 chemicals with a high concern for carcinogenesis were selected from a list of 1623 FCC as the top priority.

Recent computational methods have displayed good capacity in the prediction of toxicity outcomes, have provided content relating to chemical exposure, and have become a powerful tool that allows for the creation and administration of large genes or proteins [81]. Most gene-set analysis websites do not offer users the chance to modify their gene-set database. The ToxPanel website was introduced by Schyman et al. [82] and allows users to conduct gene-set analysis to evaluate liver and kidney injuries using activation scores based on gene-expression fold-change values. The ToxicoDB, created by Nair et al. [83], incorporates information from extensive in vitro toxicogenomic studies, including gene-expression profiles of primary human and rat hepatocytes treated with 231 potential toxins. The gathered information can eventually be used in preclinical toxicity studies and contribute to our growing understanding of negative outcomes.

To clarify connections between exposure to environmental chemicals and their mixtures and related health outcomes, effect biomarkers can be used. Zare et al.’s [84] research highlights the potential of effect biomarkers for monitoring chemical exposures in general and occupational populations, as well as the role of the adverse outcome pathway (AOP) framework and physiologically based kinetic and dynamic (PBK/D) modeling. They also provide an overview of the effect biomarkers that are currently available for this purpose.

Aguirre-Plans et al. [85] used phenotype-gene association data from DisGeNET and a nonparametric test comparing the gene expression of DILI-Concern and No-DILI-Concern drugs (per DILIrank definitions) in order to search for gene signatures in the CMap gene-expression data. These authors came to the conclusion that while combining the two features did not increase the classifiers’ quality, it did increase their robustness, as demonstrated by independent holdout tests.

Krewski et al. [86] discussed the development of the vision for toxicity testing in the twenty-first century and presented a vision for the next generation of risk science, incorporating risk-assessment methodologies appropriate for the analysis of new toxicological and exposure data, toxicity testing, exposure measurement, and risk assessment, leading to recommendations for human exposure.

Granados et al. [6] researched the aryl hydrocarbon receptor (AHR), a receptor that can identify xenobiotics and control the expression of the genes responsible for their metabolism. A total of 290 biochemical pathway metabolites, such as fatty acids, antioxidants, and uremic toxins, are regulated by the AHR through interaction. When AHR expression is reduced, abnormal aldehyde accumulation occurs. Glyceraldehyde 3-phosphate dehydrogenase expression is also decreased, which disturbs glucose homeostasis. These effects are caused by the proteome profiling of zebrafish livers using isobaric tags multiplexed quantitative proteomics [15].

## 11. Concluding Remarks

There is no doubt concerning the great advance of knowledge in each of the areas of toxicology with the use of the new omics methodologies, as well as of the implementation and use of artificial intelligence and bioinformatics. This great advance, among many others, will allow for the design of new preventive programs for the early detection of the most frequent, disabling, and deadly diseases by identifying their biomarkers, and by understanding the modes of action of xenobiotics and their relationship with the development and evolution of diseases, as well as the creation of novel, safer, and more effective medications by determining their specific targets at the genetic and molecular level.

In this new era of GT, many researchers are developing new and more complete and complex research; however, many of them are working individually in their own areas of expertise, which brings about a nonlinear and noncollaborative advance in the knowledge of the most relevant and important diseases in humans. Therefore, it would be very convenient and most pertinent for institutions, organizations, or international leaders in toxicology to design and implement a comprehensive and collaborative work plan from the methodological and analytical point of view, prioritizing the most relevant diseases, to assess and subsequently invite every expert researcher in each of the areas of GT to collaborate in multidisciplinary and multi-institutional projects with the aim of acquiring knowledge of the normal molecular processes and how they can be affected by exposure to xenobiotics and environmental stressors, in this fashion decreasing morbimortality in humans.

## Figures and Tables

**Table 1:** Relevant studies on GT in toxicology.

Omics Field	Matrix	Aim	Comment	Omics Assessment	Omics Database	Author
Proteomics	Rat spleen.	To evaluate iron oxide nanoparticles toxicity mechanism.	197 upregulated and 75 downregulated proteins, the AKT/mTOR/TFEB in splenic macrophages, a signaling route promoted autophagy and lysosomal activation.	Tandem mass tag-labeled quantitative proteomics.	Uniprot, geneontology.org, genome.jp.	Han et al., 2022 [2]
Proteomics	*Bothrops atrox* venom.	To measure changes in the plasma proteome of a model of envenomated mice.	*B. atrox* pathophysiology is caused by direct venom toxicity and indirect mechanisms derived from the tissue inflammatory response to envenomation, such as thromboinflammatory changes in fat metabolism and disturbances in the cell caused by oxidative stress and effects on pathways that regulate gene expression, survival, and cell cycle.	Shotgun proteomics analysis, LC-MS/MS, nanoelectrospray ionization.	UniProtKB/Swiss-Prot, PatternLab, DAVID.	Cavalcante et al., 2022 [3]
Proteomics	Adolescent rat amygdala.	To measure changes in the plasma proteome of a model of envenomated mice.	*B. atrox* pathophysiology is caused by direct venom toxicity and indirect mechanisms derived from the tissue inflammatory response to envenomation, such as thromboinflammatory changes in fat metabolism and disturbances in the cell caused by oxidative stress and effects on pathways that regulate gene expression, survival, and cell cycle.	LC–MS/MS analysis, electrospray ionization.	Thermo Proteome Discoverer, DAVID.	Alugubelly et al., 2021 [4]
Transcriptomics	Rat testis and liver.	To generate apical, hormone, and liver and testis toxicogenomic data following a short-term (14 day) exposure to myclobutanil to compare points of departure from regulatory guideline studies.	Biological effects point of departure from a 14-day study was sensitive apical POD across regulatory guideline studies	Testis and liver transcriptomic as RNA sequencing.	TG-GATES.	LaRocca et al., 2020 [5]
Metabolomics	Knockout Ahr−/− mice.	To analyze AHR contributions to metabolism across multiple scales from the organ to the organelle.	AHR interacts in the regulation of 290 metabolites of biochemical pathways from fatty acids, antioxidants, to uremic toxins, which demonstrates different metabolic functions that AHR performs throughout the body.	Global metabolic profiling analysis.	Pubchem, ChemRICH, Gene Expression Omnibus.	Granados et al., 2022 [6]
Metabolomics	Mice liver.	To evaluate the combinate toxicity of hexaconazole and Arsenic (As).	As modifies amino-acid-related pathways and metabolites linked to nerve disease, an insufficient supply of energy, and a functional disorder of the liver.	UPLC-MS/MS, electrospray ion, untargeted metabolomics.	BiotreeDB.	Sun et al., 2021 [7]
Metabolomics	Human plasma.	To identify plasma metabolite changes under conditions of high Pb concentration and low cognition.	20 dysregulated metabolites were found, and GO analysis revealed their significance for both normal brain function and the development of neurodegenerative illnesses such as Parkinson’s disease.	LC-MS, untargeted metabolomic analysis.	Online Human Metabolome Database.	Wang et al., 2022 [8]
Epigenomics	Buccal and peripheral blood mononuclear cells.	To standardize preprocessing data pipelines and statistical methods to detect As related DNAm signatures.	As exposure has DNA methylation signatures in individual regions and pathways also mechanisms related to As toxicity.	850 k and 450 k microarray, epigenome-wide association studies.	Kyoto Encyclopedia of Genes and Genomes.	Bozack et al., 2021 [9]
Epigenomics	Brain tissue of C57BL/6 wild-type and APP/PS mice.	To evaluate the As epigenomic alterations in pathophysiological neural changes, in particular histone methylation profile manifesting as cognitive decline.	Developmental As exposure affects histone modifications in the brain that persist into adulthood. A potential mechanism of As exposure influencing cognitive function suppressing biological processes related to neuronal development.	Chromatin immunoprecipitation and sequencing.	Database for Annotation, Visualization and Integrated Discovery (DAVID).	Fitz et al., 2022 [10]
Epigenomics	Adult whole-blood-derived DNA.	To evaluate potential DNA methylation processes and correlations between heavy metals and impairment of activities of daily living (ADL).	In older people, a higher incidence of ADL impairment was linked to higher exposures to manganese, copper, arsenic, and cadmium. Possible DNA methylation pathways for various heavy metals and possible biomarkers of ADL were revealed by epigenome-wide associations.	Human methylation EPIC bead chip array, EWAS.	Kyoto Encyclopedia of Genes and Genomes.	Xiao et al., 2022 [11]
Transcriptomic	Human colon mucosal epithelial cells (NCM460).	To examine possible outcomes and underlying processes of exposure to bisphenol S (BPS) on human colon mucosal epithelial cells (NCM460).	The gut–brain axis may become out of balance as a result of exposure to BPS, which could cause the NCM460 cells to produce inflammatory cytokines and the destruction of tight junctions.	A multiomics study.		Ao et al., 2021 [12]
Transcriptomic	The hTERT-immortalized human urothelial cell line (TRT-HU1).	To identify and validate potential candidate biomarkers for the detection, prognosis, and treatment of bladder cancer by demonstrating the gene-expression patterns and transformative changes.	Stem cell activators may be crucial in helping arsenic-exposed cells obtain a survival edge, allowing healthy epithelial cells to reprogram into a cancer stem cell phenotype, and ultimately resulting in malignant transformation.	For high-quality mapping reads to the reference genome, spliced transcripts were aligned to a reference (STAR version 2.7) software.	STAR version 2.7 is free open-source software distributed under GPLv3 license and can be downloaded from http://code.google.com/p/rna-star/ (accessed on 25 April 2023).	Shukla et al., 2022 [13]
Proteomic	Parotid, submandibular (SM) sublingual (SL) glands of rat.	To identify and validate potential candidate biomarkers for the detection, prognosis, and treatment of bladder cancer by demonstrating the gene-expression patterns and transformative changes.	Stem cell activators may be crucial in helping arsenic-exposed cells obtain a survival edge, allowing healthy epithelial cells to reprogram into a cancer stem cell phenotype, and ultimately resulting in malignant transformation.	Mass spectrometry.	networkanalyst.ca.	Cunha Nascimento et al., 2021 [14]
Proteomic	Blood of zebrafish adults (Danio rerio, AB-wild type).	To research multiplexed quantitative proteomics for zebrafish liver proteome profiling using isobaric tags.	Aldehyde dehydrogenase expression was downregulated, which caused a buildup of aldehydes, which decreased the expression of glyceraldehyde 3-phosphate dehydrogenase and disrupted glucose homeostasis.	Liquid chromatography mass spectrometry.	Gene Ontology (GO) annotation dataset was derived from DAVID (https://david.ncifcrf.gov).	Gao et al., 2022 [15]
Metabolomic	BEAS-2B noncancerous human bronchial epithelial cells.	To treat human bronchial epithelial cells with As for 6 to 24 weeks and assess spatiotemporal metabolic patterns of biomolecules, cofactors, and xenobiotics through global untargeted metabolic analysis.	In the cells treated with As^3+^ for 6 to 13 weeks, a significant suppression of all metabolites in the mitochondrial tricarboxylic acid (TCA) cycle was observed. This thorough metabolomics investigation offers fresh perspectives on the metabolic disruption caused by As and may result in more precise evidence of its role in molecular carcinogenesis.	584 molecules with known identities are included in the metabolomics collection and are referred to as “biochemicals”.	UCSC genome browser.	Fu et al., 2022 [16]

## Data Availability

Not applicable.
